# Supplementary figures and images for: Tau Transfer via Extracellular Vesicles Disturbs the Astrocytic Mitochondrial System
Source: Cells. 2023 Mar 23;12(7):985. doi: 10.3390/cells12070985 (PMC10093208; doi:10.3390/cells12070985)

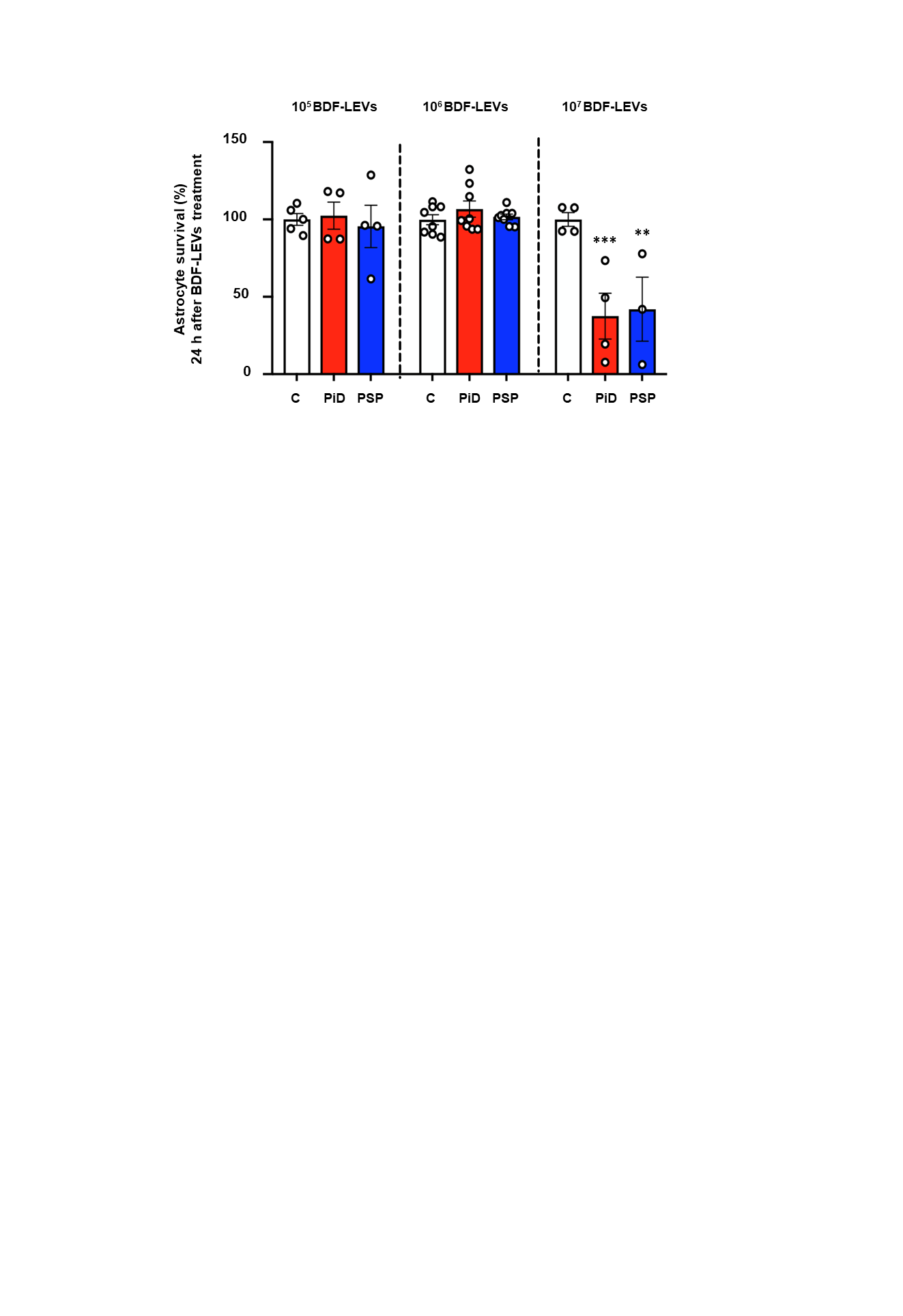

Supplement: Supplementary file 1 [file cells-12-00985-s001.zip › cells-2253573-supplementary proof done/supp tiff/Figure S1/Diapositive1.TIF]

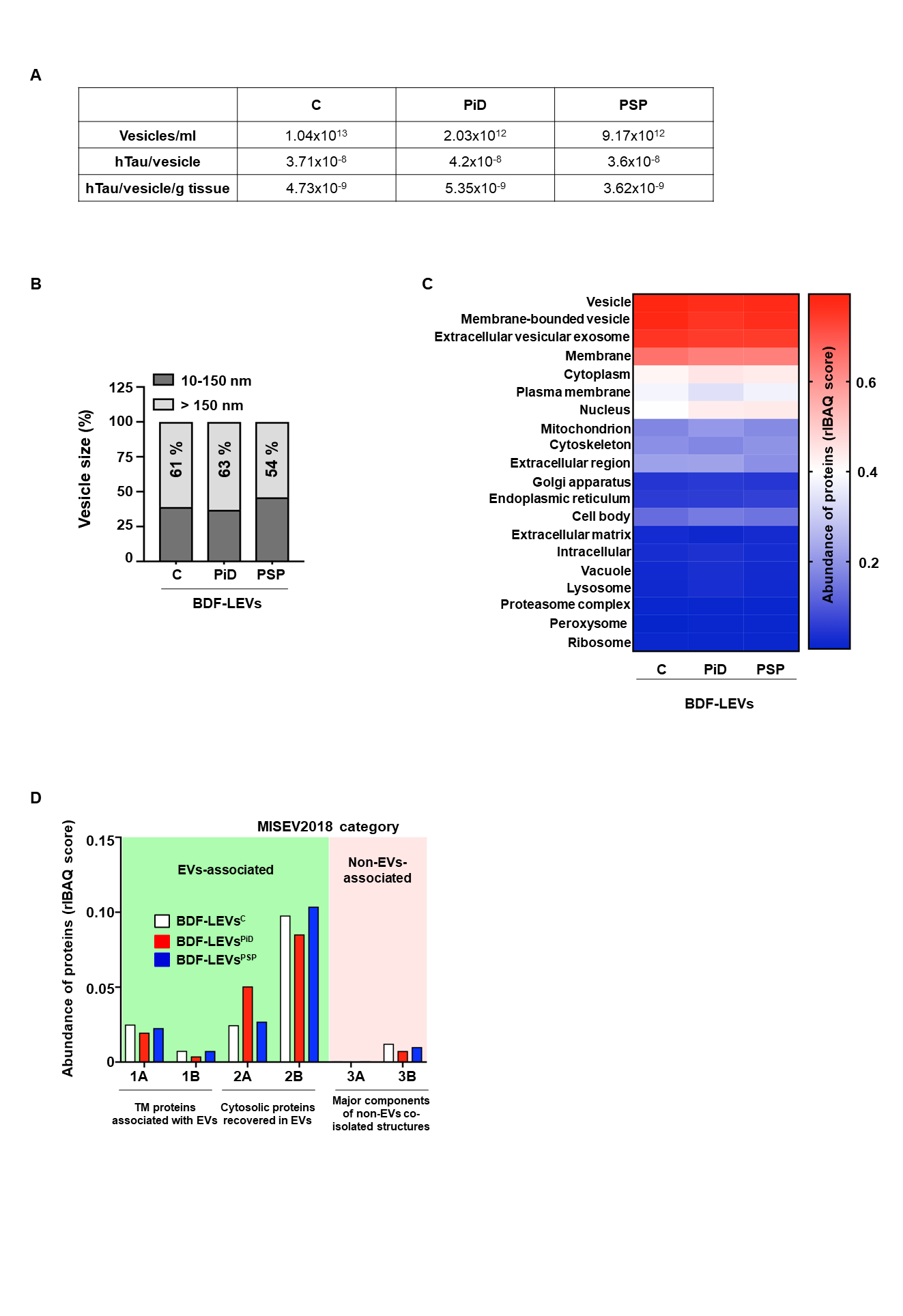

Supplement: Supplementary file 1 [file cells-12-00985-s001.zip › cells-2253573-supplementary proof done/supp tiff/Figure S2/Diapositive1.TIF]
